# Association of metabolic dysfunction-associated steatotic liver disease trajectories with incident liver cancer: a UK Biobank cohort study

**DOI:** 10.3389/fendo.2026.1851106

**Published:** 2026-06-22

**Authors:** Ryuk Jun Kwon, Yohwan Lim

**Affiliations:** 1 Family Medicine Clinic and Research Institute for Convergence of Biomedical Science and Technology, Pusan National University Yangsan Hospital, Yangsan, Republic of Korea; 2 Department of Family Medicine, Pusan National University School of Medicine, Yangsan, Republic of Korea; 3 Gochang-gun Public Health Center, Ministry of Health and Welfare, Gochang, Republic of Korea; 4 Department of Biomedical Informatics, CHA University School of Medicine, Seongnam, Republic of Korea

**Keywords:** fatty liver disease, liver cancer, MASLD, trajectory, UK Biobank

## Abstract

**Background:**

Metabolic dysfunction-associated steatotic liver disease (MASLD) is increasingly recognized as a contributor to hepatocellular carcinoma. Whether repeated assessment of MASLD provides additional prognostic information for liver cancer risk remains unclear.

**Methods:**

Using the UK Biobank, we included 12,111 participants who underwent both baseline and follow-up health screenings. MASLD was defined at each assessment as fatty liver index ≥60 plus at least one cardiometabolic risk criterion. Four trajectory groups were defined: no MASLD → no MASLD, no MASLD → MASLD, MASLD → no MASLD, and MASLD → MASLD. Cox proportional hazards models were used to estimate adjusted hazard ratios (aHR) and 95% confidence intervals (CI).

**Results:**

At baseline, 4,008 participants (33.1%) had MASLD. MASLD at period 1 was associated with a higher risk of incident liver cancer compared with no MASLD (aHR, 2.81; 95% CI, 1.20–6.58). A similar association was observed for MASLD at period 2 (aHR, 2.61; 95% CI, 1.12–6.06). In trajectory analyses, MASLD → no MASLD (aHR, 4.00; 95% CI, 1.01–15.80) and MASLD → MASLD (aHR, 3.51; 95% CI, 1.31–9.44) were associated with higher liver cancer risk than no MASLD → no MASLD.

**Conclusions:**

MASLD at either assessment was associated with higher incident liver cancer risk. Trajectory analyses suggest that persistent MASLD and prior MASLD exposure may both carry prognostic importance.

## Introduction

Metabolic dysfunction-associated steatotic liver disease (MASLD) is one of the most common chronic liver disorders worldwide, and liver cancer remains a major contributor to cancer-related mortality (1). MASLD emerged from the recent revision of the fatty liver disease definition that moved away from the older term, nonalcoholic fatty liver disease (NAFLD) (2). Although these entities overlap considerably, MASLD places greater emphasis on the presence of metabolic dysfunction rather than defining disease primarily by exclusion. As the burden of steatotic liver disease continues to rise, its contribution to hepatocellular carcinoma (HCC) has become an increasingly important clinical and public health issue (3).

Many observational studies indicate that fatty liver disease is associated with liver cancer risk. In a Swedish population-based cohort using biopsy-confirmed NAFLD, the excess cancer burden was driven largely by HCC (4). Similarly, another study reported that metabolic dysfunction-associated fatty liver disease was associated with increased risk of several site-specific cancers, including liver cancer, and that this association persisted even after additional adjustment for anthropometric measures (1).

Most prior cohort studies have defined fatty liver disease at a single baseline time point. Such an approach may be insufficient because steatotic liver disease is dynamic. It may persist, newly develop, or appear to resolve over time. A recent longitudinal study from South Korea raises the possibility that prior MASLD exposure may increase HCC risk even when the phenotype is no longer detected at a later visit (5). However, MASLD trajectories and subsequent liver cancer risk remain limited in Western population-based cohorts.

Therefore, using the UK Biobank, we examined whether MASLD status at baseline and consecutive health screening was associated with incident liver cancer and whether changes in MASLD status between these two assessments provided additional prognostic information.

## Methods

### Study population

#### UK Biobank cohort

**Figure 1:**
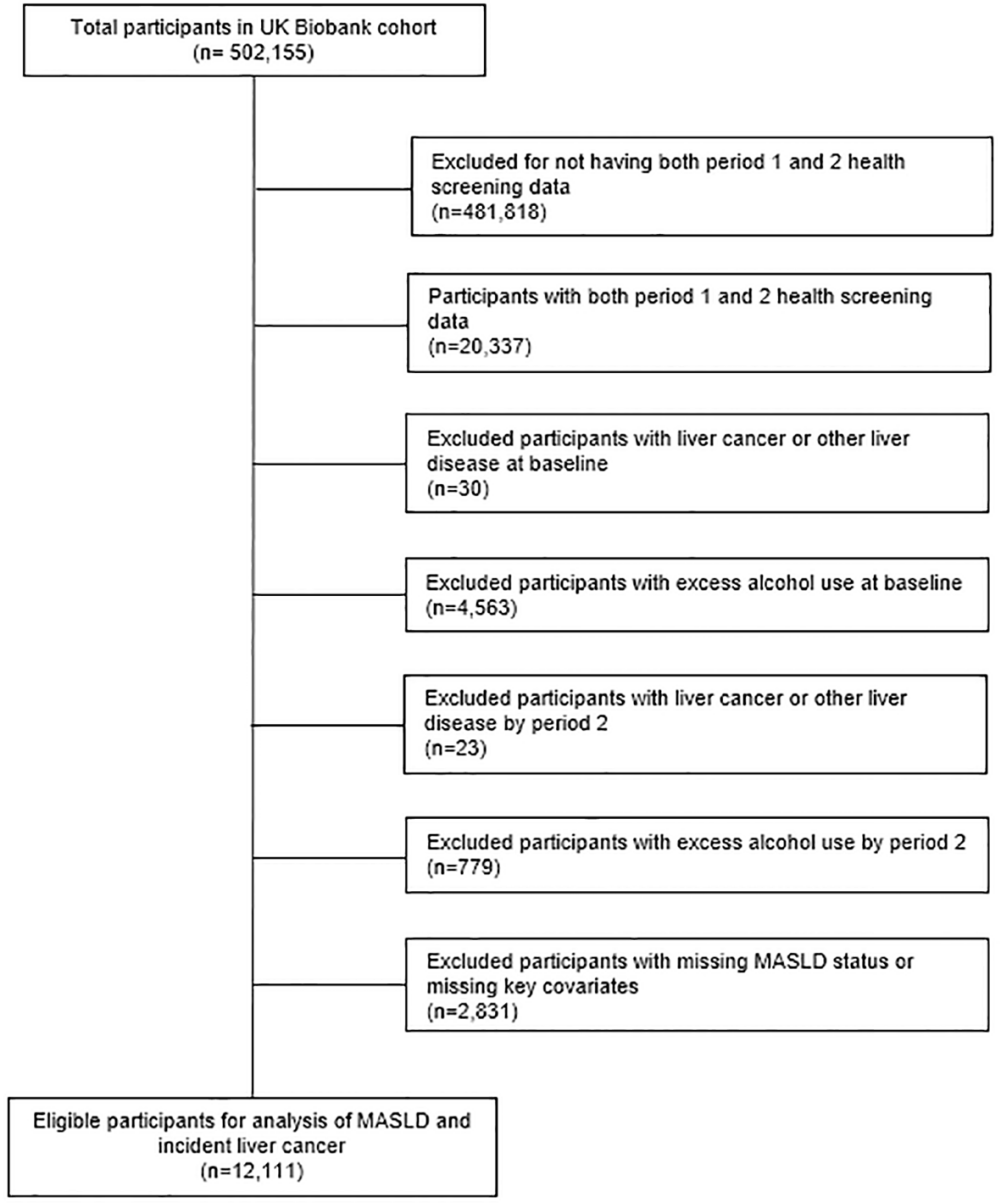
Flow diagram for the inclusion of the study population.

This study was conducted using the UK Biobank cohort. It is a prospective cohort that enrolled adults across the United Kingdom and linked baseline assessment data with consecutive health screenings, hospital inpatient records, cancer registry data, medication data, and mortality records for use in a wide range of scientific research (6). It includes approximately 500,000 participants in the United Kingdom that were recruited between 2006 and 2010. Further details of the UK Biobank cohort and data resources are available elsewhere (7). Baseline was defined as the first assessment visit (period 1), and the consecutive health screening was defined as the first repeat assessment visit (period 2). Among 20,337 participants in the analytic extract with both period 1 and 2 assessment data, participants were excluded if they had liver cancer at baseline (*n* = 4), other liver disease at baseline (*n* = 26), excess alcohol use at baseline (*n* = 4,563), liver cancer by period 2 (*n* = 0), other liver disease by period 2 (*n* = 23), excess alcohol use by period 2 (*n* = 779), missing MASLD status at baseline (*n* = 1,114), missing MASLD status at period 2 (*n* = 1,691), missing age (*n* = 0), missing sex (*n* = 0), or missing smoking status (*n* = 26). The final analytic cohort included 12,111 participants (**Figure 1**). Our study using the UK Biobank dataset obtained Research Tissue Bank approval from the North West Multi-centre Research Ethics Committee (ID: 16/NW/0274).

### Definition of MASLD

MASLD status was determined separately at periods 1 and 2. Hepatic steatosis was identified using the fatty liver index (FLI), calculated from triglycerides, gamma-glutamyl transferase (GGT), body mass index (BMI), and waist circumference (WC) (8). Triglyceride concentrations were converted from mmol/L to mg/dl before FLI calculation, and steatosis was defined as FLI ≥ 60. FLI was computed as:


FLI=e(0.953×ln(triglycerides[mg/dL])+0.139×BMI+0.718×ln(GGT)+0.053×WC−15.745)/(1+e(0.953×ln(triglycerides[mg/dL])+0.139×BMI+0.718×ln(GGT)+0.053×WC−15.745))×100

To meet the metabolic dysfunction definitions, participants were required to have at least one cardiometabolic risk criterion. These criteria were defined as overweight or obesity based on ethnicity-specific BMI thresholds (BMI ≥ 23 kg/m² in Asian participants and ≥25 kg/m² in non-Asian participants), hyperglycemia or diabetes (glucose ≥ 5.6 mmol/L or antidiabetic medication use), elevated blood pressure (systolic blood pressure ≥ 130 mmHg, diastolic blood pressure ≥ 85 mmHg, or antihypertensive medication use), and dyslipidemia (triglycerides ≥ 1.7 mmol/L, low HDL cholesterol, or lipid-lowering medication use) (9). Low HDL cholesterol was defined as < 1.0 mmol/L in men and < 1.3 mmol/L in women. Ethnicity-specific BMI thresholds were applied by classifying Asian participants separately from non-Asian participants. Participants were excluded from MASLD classification at a given assessment if they had evidence of other liver diseases recorded before the assessment date. Exclusionary liver diseases were identified from ICD-10 codes and included viral hepatitis, alcohol-related liver disease, toxic liver disease, selected cholestatic liver diseases, autoimmune hepatitis, Wilson disease, and hemochromatosis (**Supplementary Table 1**). In addition, participants reporting daily or almost daily alcohol consumption were treated as having excess alcohol exposure and were excluded from MASLD. Cirrhosis-related ICD-10 codes were identified separately.

### MASLD trajectory

MASLD trajectory was constructed using MASLD status at period 1 and 2. Four trajectory groups were defined: no MASLD to no MASLD, no MASLD to MASLD, MASLD to no MASLD, and MASLD to MASLD.

### Outcome ascertainment

The primary outcome was incident liver cancer. Liver cancer events were identified using ICD-10 code C22 from hospital inpatient records and cancer registry records. For each participant, the event date was defined as the earliest liver cancer diagnosis date across these sources. Participants with liver cancer diagnosed before the assessment date were excluded from the analysis.

### Key variable

Key variables were defined using baseline assessment at period 1. Smoking status was categorized as never, former, or current smoker. Income level was dichotomized into upper half and lower half categories based on the original household income. Alcohol consumption was defined as yes or no using self-reported drinking status and frequency. Moderate-to-vigorous physical activity (MVPA) was derived by summing the reported frequencies of moderate and vigorous physical activity and grouped as 0, 1–2, 3–4, or ≥5 times per week. Hypertension was defined as systolic blood pressure ≥140 mmHg, diastolic blood pressure ≥90 mmHg, or antihypertensive medication use. Diabetes was defined as serum glucose ≥7.0 mmol/L, HbA1c ≥48 mmol/mol, self-reported physician diagnosis, or antidiabetic medication use. Dyslipidemia was defined as triglycerides ≥1.7 mmol/L, low HDL cholesterol based on sex-specific thresholds, or lipid-lowering medication use. Charlson comorbidity index (CCI) was derived from diagnosis data and categorized as 0, 1, or ≥2. Baseline BMI category was classified using ethnicity-specific cutoffs. Among Asian participants, underweight was defined as <18.5 kg/m², normal weight as 18.5 to <23.0 kg/m², overweight as 23.0 to <25.0 kg/m², and obese as ≥25.0 kg/m². Among non-Asian participants, underweight was defined as <18.5 kg/m², normal weight as 18.5 to <25.0 kg/m², overweight as 25.0 to <30.0 kg/m², and obese as ≥30.0 kg/m². The operational definitions of routinely measured baseline covariates were adapted from previous epidemiologic studies. The present study applied these definitions to our database (10–16).

### Statistical analysis

Continuous variables were presented as mean with standard deviation, and categorical variables were presented as number with percentage. Incidence rates were calculated as the number of events per 1,000 person-years, with a 95% confidence interval (CI) derived from the Poisson distribution. Associations of MASLD status and MASLD trajectory with incident liver cancer were examined using Cox proportional hazards model and estimated adjusted hazard ratios (aHR) with 95% CI. Given the limited number of incident liver cancer events, we used a limited number of covariates to reduce model overfitting and instability of regression estimates. Model 1 was adjusted for age and sex. Model 2 was adjusted for age, sex, and smoking status. Model 3 additionally adjusted for alcohol consumption and the Charlson comorbidity index. Follow-up started at period 2 for trajectory analyses and continued until liver cancer diagnosis, death, administrative censoring, or March 31, 2023, whichever occurred first. A sensitivity analysis was additionally adjusted for BMI change between period 1 and 2. We also performed a sensitivity analysis excluding participants with cirrhosis before period 2. Cirrhosis-related ICD-10 codes (K70.3, K71.7, K74) were identified separately from the main exclusionary liver disease codes. Cumulative incidence curves were plotted according to MASLD trajectory groups. *P*-value < 0.05 was considered as statistically significant. Statistical analyses were conducted using SAS version 9.4 and RStudio version 7.2 (RStudio, New York, USA).

## Results

### Participant characteristics

A total of 12,111 participants were included in the analytic cohort, with a mean age of 56.8 (7.5) years, and 5,631 participants (46.5%) were men. The mean interval between period 1 and 2 was 4.30 years. At period 1, 8,103 participants (66.9%) were classified as having no MASLD and 4,008 participants (33.1%) as having MASLD. Compared with participants without MASLD, those with MASLD were slightly older, were more likely to be men, and had a less favorable cardiometabolic profile, including higher frequencies of hypertension, diabetes, dyslipidemia, and obesity. Additional baseline characteristics are summarized in **Table 1**. Included and excluded participants are compared in **Supplementary Table 2**, and the cohort selection process is shown in **Supplementary Table 3**.

**Table 1:** Baseline characteristics of study population in period 1.

	Total	No MASLD	MASLD
Characteristic	(*n* = 12,111)	(*n* = 8,103)	(*n* = 4,008)
Age, years	56.8 (7.5)	56.5 (7.5)	57.4 (7.4)
Sex, *n* (%)			
Men	5,631 (46.5)	2,987 (36.9)	2,644 (66.0)
Women	6,480 (53.5)	5,116 (63.1)	1,364 (34.0)
BMI, *n* (%)			
Underweight	53 (0.4)	53 (0.7)	0 (0.0)
Normal	4,447 (36.7)	4,377 (54.0)	70 (1.7)
Overweight	5,040 (41.6)	3,353 (41.4)	1,687 (42.1)
Obese	2,571 (21.2)	320 (3.9)	2,251 (56.2)
Hypertension, *n* (%)			
No	5,638 (46.6)	4,443 (54.8)	1,195 (29.8)
Yes	6,473 (53.4)	3,660 (45.2)	2,813 (70.2)
Diabetes, *n* (%)			
No	11,593 (95.7)	7,926 (97.8)	3,667 (91.5)
Yes	518 (4.3)	177 (2.2)	341 (8.5)
Dyslipidemia, *n* (%)			
No	6,405 (52.9)	5,590 (69.0)	815 (20.3)
Yes	5,706 (47.1)	2,513 (31.0)	3,193 (79.7)
Charlson comorbidity index, *n* (%)			
0	10,736 (88.6)	7,343 (90.6)	3,393 (84.7)
1	604 (5.0)	351 (4.3)	253 (6.3)
≥2	771 (6.4)	409 (5.0)	362 (9.0)
Cigarette smoking, *n* (%)			
Current smoker	683 (5.6)	412 (5.1)	271 (6.8)
Former smoker	3,703 (30.6)	2,184 (27.0)	1,519 (37.9)
Never smoker	7,725 (63.8)	5,507 (68.0)	2,218 (55.3)
Alcohol consumption, *n* (%)			
Yes	9,709 (80.2)	6,504 (80.3)	3,205 (80.0)
No	2,397 (19.8)	1,594 (19.7)	803 (20.0)
MVPA, *n* (%)			
Physically inactive	1,368 (11.4)	763 (9.5)	605 (15.3)
1–2 times/week	1,832 (15.3)	1,168 (14.5)	664 (16.8)
3–4 times/week	2,264 (18.9)	1,527 (19.0)	737 (18.7)
≥ 5 times/week	6,512 (54.4)	4,574 (56.9)	1,938 (49.1)

Continuous variables are presented as mean (standard deviation), and categorical variables are presented as n (%). Percentages for alcohol consumption and MVPA were calculated among participants with non-missing data. MASLD, metabolic dysfunction-associated steatotic liver disease; BMI, body mass index; MVPA, moderate-to-vigorous physical activity.

### MASLD status in period 1 and 2 with incident liver cancer

During follow-up, 24 incident liver cancer events occurred. In period 1 analyses, liver cancer developed in 10 of 8,103 participants without MASLD and in 14 of 4,008 participants with MASLD. The incidence rate was 0.09 per 1,000 person-years in the no-MASLD group and 0.25 per 1,000 person-years in the MASLD group. MASLD at period 1 was associated with higher liver cancer risk in Model 3 (aHR, 2.81; 95% CI, 1.20–6.58). A similar pattern was observed for MASLD at period 2. MASLD at period 2 was associated with higher liver cancer risk in Model 3 (aHR, 2.61; 95% CI, 1.12–6.06) (**Table 2**).

**Table 2:** Incidence rates and hazard ratios for incident liver cancer according to MASLD status at period 1 and 2.

MASLD status	Event/Total	PY	IR per 1000 PY (95% CI)	Model 1 aHR^a^ (95% CI)	P for trend	Model 2 aHR^b^ (95% CI)	P for trend	Model 3 aHR^c^ (95% CI)	P for trend
Period 1									
No MASLD	10/8,103	115,762	0.09 (0.04–0.16)	1.00 (Ref)	0.0136	1.00 (Ref)	0.0129	1.00 (Ref)	0.0174
MASLD	14/4,008	56,687	0.25 (0.14–0.41)	2.90 (1.24–6.74)		2.93 (1.26–6.86)		2.81 (1.20–6.58)	
Period 2									
No MASLD	10/7,922	78,951	0.13 (0.06–0.23)	1.00 (Ref)	0.0213	1.00 (Ref)	0.0199	1.00 (Ref)	0.0259
MASLD	14/4,189	41,401	0.34 (0.18–0.57)	2.67 (1.16–6.16)		2.72 (1.17–6.30)		2.61 (1.12–6.06)	

MASLD status was defined at period 1 and 2. MASLD was defined as FLI ≥60 with at least one cardiometabolic risk criterion, after excluding participants with excess alcohol use or other liver diseases. Cardiometabolic risk criteria included overweight/obesity based on ethnicity-specific BMI thresholds, elevated glucose or diabetes, elevated blood pressure or antihypertensive medication use, and elevated triglycerides, low HDL cholesterol, or lipid-lowering medication use.

**^a^**Model 1 was adjusted for age and sex.

**^b^**Model 2 was adjusted for age, sex, and smoking status.

**^c^**Model 3 was adjusted for age, sex, smoking status, alcohol consumption, and Charlson comorbidity index.

Acronyms: MASLD, metabolic dysfunction-associated steatotic liver disease; FLI, fatty liver index; PY, person-years; IR, incidence rate; adjusted HR, hazard ratio; CI, confidence interval.

### MASLD trajectory and incident liver cancer

**Figure 2:**
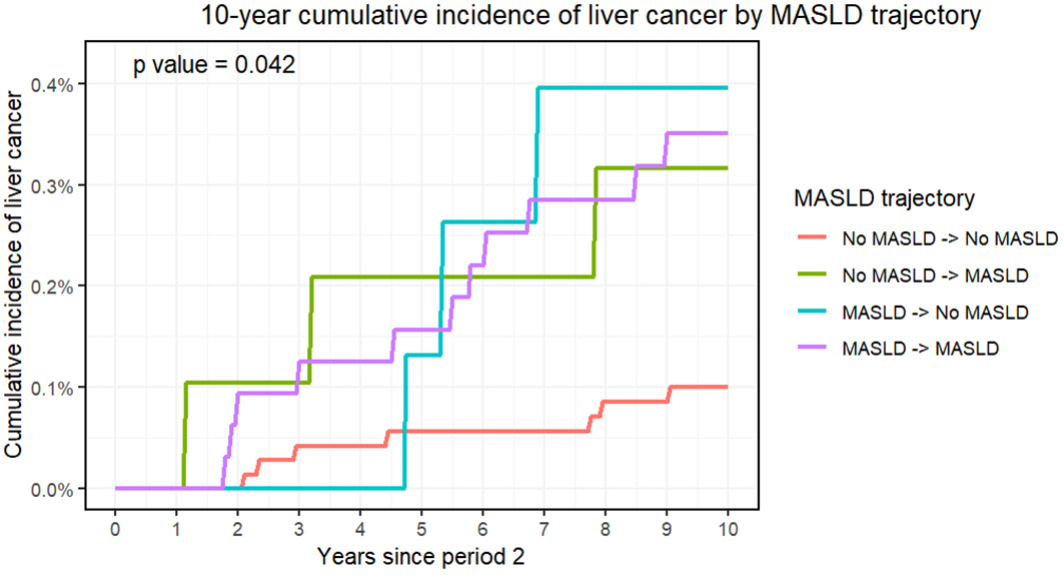
10-year cumulative incidence of liver cancer by MASLD trajectory. MASLD trajectory was defined using MASLD status at period 1 (baseline assessment) and period 2 (consecutive health screening) and classified into four groups: no MASLD → no MASLD, no MASLD → MASLD, MASLD → no MASLD, and MASLD → MASLD. The figure shows cumulative incidence for incident liver cancer followed from period 2. The p value shown in the figure was derived from Cox proportional hazards models adjusted for age, sex, smoking status, alcohol consumption, and Charlson comorbidity index. Acronym: MASLD, Metabolic dysfunction-associated steatotic liver disease.

When participants were classified according to MASLD trajectory from period 1 to 2, the lowest liver cancer incidence rate was observed in the no MASLD → no MASLD group (0.10 per 1,000 person-years). Higher incidence rates were observed in the no MASLD → MASLD group (0.31 per 1,000 person-years), MASLD → no MASLD group (0.39 per 1,000 person-years), and MASLD → MASLD group (0.35 per 1,000 person-years). In Model 3, compared with no MASLD → no MASLD, the aHR were 3.28 (95% CI, 0.84–12.81) for no MASLD → MASLD, 4.00 (95% CI, 1.01–15.80) for MASLD → no MASLD, and 3.51 (95% CI, 1.31–9.44) for MASLD → MASLD (**Table 3**). The estimated 10-year cumulative incidence was 0.10% for no MASLD → no MASLD, 0.32% for no MASLD → MASLD, 0.40% for MASLD → no MASLD, and 0.35% for MASLD → MASLD (*p* = 0.042) (**Figure 2**).

**Table 3:** Incidence rates and hazard ratios for incident liver cancer according to MASLD trajectory from period 1 to 2.

MASLD trajectory	Event/Total	PY	IR per 1000 PY (95% CI)	Model 1 aHR^a^ (95% CI)	P for trend	Model 2 aHR ^b^ (95% CI)	P for trend	Model 3 aHR ^c^ (95% CI)	P for trend
No MASLD → no MASLD	7/7,140	71,250	0.10 (0.04–0.20)	1.00 (Ref)	0.0089	1.00 (Ref)	0.0083	1.00 (Ref)	0.0116
No MASLD → MASLD	3/963	9,635	0.31 (0.06–0.91)	3.25 (0.84–12.64)		3.31 (0.85–12.92)		3.28 (0.84–12.81)	
MASLD → no MASLD	3/782	7,700	0.39 (0.08–1.14)	4.03 (1.02–15.87)		4.06 (1.03–16.02)		4.00 (1.01–15.80)	
MASLD → MASLD	11/3,226	31,766	0.35 (0.17–0.62)	3.63 (1.36–9.67)		3.70 (1.38–9.91)		3.51 (1.31–9.44)	

MASLD trajectory was defined using MASLD status at period 1 and 2. Participants were classified into four groups: no MASLD → no MASLD, no MASLD → MASLD, MASLD → no MASLD, and MASLD → MASLD. MASLD was defined as FLI ≥60 with at least one cardiometabolic risk criterion, after excluding participants with excess alcohol use or other liver diseases.

a Model 1 was adjusted for age and sex.

b Model 2 was adjusted for age, sex, and smoking status.

c Model 3 was adjusted for age, sex, smoking status, alcohol consumption, and Charlson comorbidity index.

Acronyms: MASLD, metabolic dysfunction-associated steatotic liver disease; FLI, fatty liver index; PY, person-years; IR, incidence rate; aHR, adjusted hazard ratio; CI, confidence interval.

In the sensitivity analysis, additionally adjusted for BMI change between period 1 and 2, the direction of association was generally consistent, and persistent MASLD remained associated with incident liver cancer (aHR, 3.70; 95% CI, 1.38–9.93). BMI change itself was not independently associated with incident liver cancer (**Supplementary Table 4**). After excluding participants with cirrhosis before period 2, the associations were generally consistent with the main analysis (**Supplementary Table 5**).

## Discussion

Using consecutive health screenings, MASLD identified that either baseline or the consecutive screening was associated with a higher incidence of liver cancer. In addition, trajectory-based analyses suggested that the excess risk was not limited to persistent MASLD. Participants with persistent MASLD and those who changed from MASLD to no MASLD both showed significantly higher risks than those who remained free of MASLD. Therefore, our findings suggest that both current MASLD status and prior MASLD exposure may be associated with liver cancer risk.

These observations are broadly consistent with prior studies with HCC. Recent reviews have emphasized that NAFLD is becoming an increasingly important component of the global burden, including cardiovascular disease, dementia, and HCC (3, 17, 18). In a Swedish population-based cohort, cancer risk for biopsy-confirmed NAFLD was driven largely by HCC, with substantially higher HCC incidence than in matched population controls (4). Another observational study also found that fatty liver disease was associated with increased risk of several site-specific cancers, including liver cancer, and that the association with liver cancer remained even after additional adjustment for body size measures (1).

Our study involving longitudinal exposure assessment is also notable. Most observational studies have relied on a single baseline definition of fatty liver disease, whereas our analysis evaluated change in MASLD status over time. This approach is supported by a recent study from Jeong et al. that focused on evolutionary changes in MASLD with HCC risk among South Koreans (5). Our findings are consistent with this study while extending this association to a population from the UK.

The elevated risk seen in the MASLD → no MASLD group needs explanation. One possible explanation is that improvement in the steatotic phenotype does not necessarily reverse cumulative hepatic injury already established before the second assessment. Residual fibrosis or fibrotic remodeling may persist despite improvement in steatosis, and fibrosis is a key determinant of adverse liver-related outcomes and an important substrate for hepatocarcinogenesis. In addition, chronic inflammatory signaling and stellate cell activation may continue to promote fibro-carcinogenic pathways even after overt MASLD is no longer detectable (19–22). This interpretation is compatible with previous studies in which resolved MASLD still carried excess HCC risk (4, 5).

Another important consideration is that FLI was developed as a population-level surrogate marker of hepatic steatosis rather than as a direct longitudinal imaging or histologic measure (8). Because FLI includes BMI, waist circumference, triglycerides, and GGT, changes in metabolic variables may change FLI classification even when intrahepatic lipid burden does not change proportionally. Thus, participants classified as MASLD → no MASLD may represent a heterogeneous group, including those with true improvement in steatosis and those with progression to more advanced fibrotic disease in which steatosis becomes less apparent. This distinction is important because fibrosis stage is a major predictor of adverse liver-related outcomes in fatty liver disease (23). Nevertheless, in our sensitivity analysis additionally adjusted for BMI change, persistent MASLD remained associated with incident liver cancer, while BMI change itself was not independently associated with incident liver cancer. These findings partially address the role of weight change, but they do not eliminate the possibility of residual exposure misclassification (24).

This study has several limitations. First, MASLD was defined using the FLI and metabolic criteria rather than imaging, elastography, or histology, so exposure misclassification can happen. Second, liver cancer events were few, which limited statistical power and likely contributed to the wide confidence intervals. This small number of events also increased the risk of model overfitting and unstable Cox regression estimates. Therefore, we used restricted covariate adjustment models and interpreted the adjusted estimates cautiously. Third, we did not directly measure fibrosis stage, which is a key determinant of HCC risk in fatty liver disease. Although a sensitivity analysis excluding participants with cirrhosis before period 2 showed generally consistent results, residual confounding by undiagnosed cirrhosis or fibrosis may remain (23). In addition, non-invasive fibrosis scores such as FIB-4 or NFS could not be consistently incorporated into the primary models due to insufficient variables in our analytic dataset, and adding additional fibrosis-related covariates would further increase model instability given the limited number of liver cancer events. Therefore, residual confounding by unmeasured fibrosis or fibrotic remodeling cannot be excluded. Fourth, residual confounding cannot be excluded, including diet or changes in metabolic risk factors between assessments. Fifth, we defined liver disease only using hospital inpatient ICD-10 diagnoses, which likely captured relatively more severe cases and may have underestimated the total burden. However, ICD-10 codes have been widely used in previous epidemiologic studies (25–31). Sixth, competing-risk models were not performed because of the limited number of liver cancer events.

## Conclusion

MASLD at baseline and at follow-up assessment was associated with a higher risk of incident liver cancer, and trajectory analyses suggested that both persistent MASLD and prior MASLD exposure may carry prognostic importance. Repeated assessment of MASLD may help identify individuals who remain at elevated risk for liver cancer even when the phenotype appears to improve over time.

## Data Availability

The data used in this study are available from the UK Biobank (https://www.ukbiobank.ac.uk/enable-your-research/apply-for-access). As restrictions apply to the availability of these data, which were used under license for the current study, the authors cannot publicly share these data. This research has been conducted using the UK Biobank Resource under Application Number 106954. Requests to access these datasets should be directed to https://www.ukbiobank.ac.uk/about-our-data/.
