# Supplementary material for: Association of metabolic dysfunction-associated steatotic liver disease trajectories with incident liver cancer: a UK Biobank cohort study
Source: Front Endocrinol (Lausanne). 2026 Jun 22;17:1851106. doi: 10.3389/fendo.2026.1851106 (PMC13333405; doi:10.3389/fendo.2026.1851106)
Supplement: Supplementary file 1 [file Table1.docx]

**Supplementary Material**

**“Association of Metabolic Dysfunction-Associated Steatotic Liver Disease Trajectories With Incident Liver Cancer: A UK Biobank Cohort Study”**

**Table S1.** List of liver diseases by ICD-10 codes

**Table S2.** Comparison of included and excluded participants

**Table S3.** Cohort selection process

**Table S4.** Sensitivity analysis additionally adjusted for BMI change

**Table S5.** Sensitivity analysis excluding participants with cirrhosis before period 2

**Table S1.** List of liver diseases by ICD-10 codes

| **Liver disease** | **ICD-10 codes** |
| --- | --- |
| Viral hepatitis | B15–B19 |
| Alcohol-related liver disease | K70 |
| Toxic liver disease | K71 |
| Cholestatic liver diseases | K74.3–K74.5 |
| Autoimmune hepatitis | K75.4 |
| Wilson disease | E83.0 |
| Hemochromatosis | E83.1 |

Acronyms: ICD-10, International Statistical Classification of Diseases and Related Health Problems 10th Revision.

**Table S2.** Comparison of included and excluded participants

| **Characteristic** | **Excluded from analytic cohort** | **Included in analytic cohort** |
| --- | --- | --- |
| Age, years | 57.6 (7.3) | 56.8 (7.5) |
| BMI, kg/m² | 26.7 (4.4) | 27.0 (4.6) |
| **Sex, n (%)** |  |  |
| Women | 3,924 (47.7) | 6,480 (53.5) |
| Men | 4,302 (52.3) | 5,631 (46.5) |
| **Smoking status, n (%)** |  |  |
| Missing | 49 (0.6) | 0 (0.0) |
| Never smoker | 4,240 (51.5) | 7,725 (63.8) |
| Former smoker | 3,339 (40.6) | 3,703 (30.6) |
| Current smoker | 598 (7.3) | 683 (5.6) |

The included group represents the final analytic cohort after revised eligibility criteria. Continuous variables are presented as mean (standard deviation), and categorical variables are presented as n (%). BMI, body mass index.

**Table S3.** Cohort selection process

| **Step** | **Excluded, n** | **Remaining, n** |
| --- | --- | --- |
| Start population in analytic extract | — | 20,337 |
| Missing baseline assessment date | 0 | 20,337 |
| Missing repeat assessment date | 0 | 20,337 |
| Liver cancer at baseline | 4 | 20,333 |
| Other liver disease at baseline | 26 | 20,307 |
| Excess alcohol use at baseline | 4,563 | 15,744 |
| Liver cancer by repeat period | 0 | 15,744 |
| Other liver disease by repeat period | 23 | 15,721 |
| Excess alcohol use by repeat period | 779 | 14,942 |
| Missing MASLD status at baseline | 1,114 | 13,828 |
| Missing MASLD status at repeat period | 1,691 | 12,137 |
| Missing age | 0 | 12,137 |
| Missing sex | 0 | 12,137 |
| Missing smoking status | 26 | 12,111 |
| Final analytic cohort | — | 12,111 |

Acronym: MASLD, metabolic dysfunction-associated steatotic liver disease.

**Table S4.** Sensitivity analysis additionally adjusted for BMI change

| **MASLD trajectory** | **aHR (95% CI)** | **P value** |
| --- | --- | --- |
| No MASLD → no MASLD | 1.00 (Ref) | — |
| No MASLD → MASLD | 3.45 (0.86–13.85) | 0.081 |
| MASLD → no MASLD | 3.81 (0.89–16.37) | 0.072 |
| MASLD → MASLD | 3.70 (1.38–9.93) | 0.009 |
| BMI change, per 1 kg/m² increase | 0.97 (0.79–1.20) | 0.793 |

The model was adjusted for MASLD trajectory, age, sex, smoking status, and BMI change between period 1 and 2. BMI change was calculated as BMI at period 2 minus BMI at period 1.

Acronym: MASLD, metabolic dysfunction-associated steatotic liver disease; BMI, body mass index; aHR, adjusted hazard ratio; CI, confidence interval.

**Table S5.** Sensitivity analysis excluding participants with cirrhosis before period 2

| **MASLD trajectory** | **Event/Total** | **PY** | **IR per 1000 PY (95% CI)** | **Model 1 aHR^a^**  **(95% CI)** | **P for trend** | **Model 2 aHR ^b^**  **(95% CI)** | **P for trend** | **Model 3 aHR ^c^**  **(95% CI)** | **P for trend** |
| --- | --- | --- | --- | --- | --- | --- | --- | --- | --- |
| No MASLD → no MASLD | 7/7139 | 71,240 | 0.10 (0.04–0.20) | 1.00 (Ref) | 0.0144 | 1.00 (Ref) | 0.0143 | 1.00 (Ref) | 0.0195 |
| No MASLD → MASLD | 3/963 | 9,635 | 0.31 (0.06–0.91) | 3.29 (0.85–12.79) |  | 3.33 (0.85–13.00) |  | 3.31 (0.85–12.93) |  |
| MASLD → no MASLD | 3/781 | 7,691 | 0.39 (0.08–1.14) | 4.13 (1.05–16.28) |  | 4.13 (1.05–16.30) |  | 4.04 (1.02–15.98) |  |
| MASLD → MASLD | 10/3222 | 31,734 | 0.32 (0.15–0.58) | 3.39 (1.25–9.21) |  | 3.42 (1.25–9.34) |  | 3.25 (1.19–8.92) |  |

MASLD trajectory was defined using MASLD status at period 1 and 2. Participants were classified into four groups: no MASLD → no MASLD, no MASLD → MASLD, MASLD → no MASLD, and MASLD → MASLD. MASLD was defined as FLI ≥60 with at least one cardiometabolic risk criterion, after excluding participants with excess alcohol use or other liver diseases.

**^a^**Model 1 was adjusted for age and sex.

**^b^**Model 2 was adjusted for age, sex, and smoking status.

**^c^**Model 3 was adjusted for age, sex, smoking status, alcohol consumption, and Charlson comorbidity index.

Acronyms: MASLD, metabolic dysfunction-associated steatotic liver disease; FLI, fatty liver index; PY, person-years; IR, incidence rate; aHR, adjusted hazard ratio; CI, confidence interval.
